# Current and Emerging Evidence for Toll-Like Receptor Activation in Sjögren's Syndrome

**DOI:** 10.1155/2018/1246818

**Published:** 2018-12-20

**Authors:** Jeremy Kiripolsky, Jill M. Kramer

**Affiliations:** ^1^Department of Oral Biology, School of Dental Medicine, State University of New York at Buffalo, Buffalo, NY 14214, USA; ^2^Department of Oral Diagnostic Sciences, School of Dental Medicine, State University of New York at Buffalo, Buffalo, NY 14214, USA

## Abstract

While the importance of Toll-like receptor (TLR) signaling is well established in many autoimmune diseases, the role of TLR activation in Sjögren's syndrome (SS) is poorly understood. Studies in mice and humans reveal that TLRs are potent mediators of inflammation in SS. TLRs are expressed and functional in salivary tissue, and TLRs in peripheral blood cells of SS patients are also upregulated and hyperresponsive to ligation. In this review, we will detail observations in mouse models regarding the importance of TLR activation in both local and systemic disease. We will then discuss studies in SS patients that provide evidence of the importance of TLR-mediated signaling in disease. While the ligands that activate TLRs in the context of SS are unknown, emerging data suggest that damage-associated molecular patterns (DAMPs) may be significant drivers of the chronic and unremitting inflammation that is characteristic of SS. We will discuss putative DAMPs that may be of clinical significance in disease. Therapies that target TLR signaling cascades will likely reduce both exocrine-specific and systemic manifestations of SS.

## 1. Introduction

Toll-like receptors (TLRs) constitute a class of pattern recognition receptors that are expressed ubiquitously [1, 2]. TLRs are ancient membrane-spanning proteins that recognize both pathogen-associated molecular patterns (PAMPs) derived from microorganisms and endogenous mediators of inflammation, termed danger-associated molecular patterns (DAMPs) [3]. TLR activation leads to recruitment of adaptor proteins within the cytosol that culminates in signal transduction. Ultimately, activation of these pathways results in the transcription of genes involved in inflammation, immune regulation, cell survival, and proliferation [3]. Although TLRs were initially thought to be important only in host defense, more recent work demonstrates a critical role for TLRs in autoimmunity [4]. While TLR signaling is required for several different autoimmune diseases, the contribution of TLR signaling to Sjögren's syndrome (SS) initiation and progression remains poorly understood [4, 5].

SS occurs in 2 forms: primary (pSS) and secondary (sSS). In pSS, SS is the sole autoimmune disease present. This is in contrast to sSS, where the disease is observed in conjunction with another autoimmune connective tissue disease [6]. In both forms of SS, salivary and lacrimal tissues are targeted by the innate and adaptive immune systems. The disease is characterized by lymphocytic infiltration of exocrine tissues along with salivary and lacrimal hypofunction [6, 7]. Loss of salivary flow results in patient discomfort, tooth decay, and difficulty in speaking and eating [8, 9]. Patients may also experience keratoconjunctivitis sicca or inflammation of the eyes as a result of dryness that often leads to ocular discomfort and impaired vision [10]. In addition, pSS patients exhibit systemic complications including hypergammaglobulinemia, fatigue, and lymphoma [6, 7]. The diagnostic criteria for SS were revised many times since the disease was initially described [11], and the current criteria include both serologic and glandular disease assessments [12]. As many as four million Americans are currently living with SS [13]. Despite its prevalence, the disease etiology is poorly understood and there is currently no known cure for SS. Therefore, understanding the underlying pathways and networks that mediate SS is crucial in order to develop targeted treatments.

We will discuss the findings that suggest a crucial role for TLR activation in SS pathogenesis. First, we will review data from several different SS mouse models that demonstrate the importance of TLRs in SS initiation and progression. Second, we will examine evidence showing dysregulation of TLR signaling in exocrine tissue and in peripheral blood mononuclear cells (PBMCs) derived from SS patients. Third, we will discuss the potential TLR ligands that may mediate chronic inflammation in disease. Targeted therapies that modulate TLR signaling will likely be efficacious in mitigating both exocrine-specific and systemic disease manifestations.

## 2. Mouse Models of pSS Reveal the Importance of TLRs in Disease

While studies in SS patients are crucial to understand disease pathogenesis, the exclusive use of human patients to study SS has several challenges, as disease development is insidious and often goes undiagnosed for several years [13]. Therefore, it is difficult to study early disease events in SS patients. Moreover, there is considerable disease heterogeneity in humans [14–16]. SS mouse models are invaluable tools that facilitate the identification of underlying disease mechanisms, as these display similar disease manifestations to humans, and are well characterized in terms of the disease progression [17–19]. Moreover, SS occurs in an accelerated timeline compared to humans. Finally, use of mouse models allows testing of therapeutics that is considerably more difficult to perform in humans [17, 18]. In the following section, we will discuss findings in mouse models that illustrate the importance of TLR activation in disease.

### 2.1. Spontaneous Development of SS: NOD/Lt and NOD-Related Strains

One of the earliest SS models described was the nonobese diabetic strain (NOD/Lt). Initially characterized as a model of type I diabetes [20], it was later found that female NOD/Lt mice spontaneously develop lymphocytic infiltration and loss of salivary flow at about 16 weeks of age [21]. The NOD/Lt strain is one of the best characterized for the study of sSS [22, 23]. Studies in submandibular gland (SMG) tissue from female NOD/Lt animals found that *TLR1*, *TLR2*, *TLR4*, and *TLR9* were increased at pre-clinical and clinical stages when compared to a pre-disease time point [24]. In addition, myeloid differentiation primary response 88 (*MyD88*), a key adaptor used in both TLR and IL-1R family member signaling [1], was elevated at both disease time points as well. This heightened expression coincided with increasing lymphocytic infiltration in the SMG, suggesting that TLR activation may contribute to the recruitment of lymphocytes to the salivary gland [24].

To determine whether TLR inhibition may be an efficacious therapeutic, female NOD/Lt mice were given chloroquine prior to disease development until 16 weeks of age (clinical disease age). Chloroquine prevents endosomal acidification, a process that is required for TLR3, TLR7, TLR8, and TLR9 signaling [25–27]. Consistent with previous studies in lupus models, treated animals displayed reduced expression of *TLR1*, *TLR2*, *TLR3*, *TLR4*, and *TLR9* in salivary tissue [24]. In addition, chloroquine decreased sialadenitis [24]. These studies suggest that TLR-mediated signaling is important for salivary-related disease manifestations in SS and inhibition of such may be of therapeutic benefit.

Additional studies in NOD/Lt mice established the importance of TLR9 activation in SS. TLR9 ligation results in phosphorylation of p38 MAPK [28]. The kinetics of TLR9 expression and activation were examined in PBMCs isolated from NOD/Lt mice at pre-disease (4-5 weeks of age), pre-clinical (8–10 weeks), and clinical disease time points (15 weeks of age) [29]. The authors found that the percentage of cells coexpressing TLR9 and phosphorylated p38 MAPK was highest at 8 weeks of age. Moreover, expression of both proteins was higher in NOD/Lt animals as compared to controls at the 5-, 8-, and 10-week time points [29]. These data suggest that TLR9 activation may occur relatively early in disease. In a separate study, salivary flow and inflammatory mediators were evaluated in NOD mice treated with TLR9 agonists [30]. Significantly, TLR9 activation resulted in increased saliva production and decreased inflammatory mediators in salivary tissue [30]. Thus, TLR9 signaling may play a protective role in SS, similar to lupus [31], and activation of TLR9-dependent pathways may be a novel therapeutic strategy in SS.

While the NOD/Lt strain is valuable in the study of sSS, the use of pSS mouse models is important to facilitate identification of SS-specific disease mechanisms, particularly in the periphery. To this end, the C57BL/6.NOD-*Aec1Aec2* pSS strain was generated from the NOD/Lt strain. C57BL/6.NOD-*Aec1Aec2* mice have two genomic regions from NOD/Lt mice referred to as *Idd* susceptibility loci (called *Idd3* and *Idd5*) that contribute to the development of SS [32]. C57BL/6.NOD-*Aec1Aec2* mice recapitulate many aspects of human disease, including loss of salivary and tear flow, presence of autoantibodies, and glandular lymphocytic infiltrates [32]. Microarray studies performed on transcripts derived from salivary tissue of C57BL/6.NOD-*Aec1Aec2* mice at a pre-disease time point showed upregulation of genes involved in *TLR* signaling pathways [33]. Specifically, *TLR3* and *TLR7* were elevated, as well as many downstream signaling intermediates such as *TRAF6*, *interferon regulatory factor 5* (*IRF5*), and *IRF7* [33]. Thus, studies in this model provide corroborative evidence that endosomal TLR signaling cascades are dysregulated early in disease.

The NOD.B10Sn-*H2^b^* (NOD.B10) mouse model is also valuable for the study of pSS. NOD.B10 mice were derived from the NOD/Lt strain by replacing the major histocompatibility locus with that from the C57BL/10 strain [34]. The resulting congenic animals develop pSS but are resistant to type I diabetes. NOD.B10 mice share many similarities with pSS patients. They have a strong female disease predilection and exhibit spontaneous disease development. Moreover, they display antinuclear autoantibodies, lymphocytic infiltrates in salivary and lacrimal tissues, and loss of salivary flow [19, 35]. Recent work demonstrates that MyD88 plays a crucial role in the development of pSS. Specifically, NOD.B10 females that lack *MyD88* (NOD.B10*^MyD88−/−^*) were protected from loss of salivary flow and demonstrate reduced prevalence of lymphocytic infiltration in the lacrimal and submandibular glands compared to the parental strain [36]. NOD.B10*^MyD88−/−^* mice were also protected from extraglandular disease manifestations. These animals displayed a decrease in lymphocytic infiltrates in both the lung and kidney compared to NOD.B10 animals despite similar splenic T and B cell populations [36]. Finally, NOD.B10*^MyD88−/−^* mice exhibited diminished total and autoreactive antibodies as compared to the NOD.B10 parental strain [36]. Of note, since both TLRs and IL-1R family signaling are dependent on MyD88, further studies are needed to determine the specific MyD88-dependent pathways that are required for disease pathogenesis. Altogether, these studies provide compelling evidence that TLRs play a crucial role in disease in NOD mice.

### 2.2. TLR Agonists Induce SS in Healthy and SS-Prone Mice

Additional studies demonstrate that activation of TLRs in both animals with a genetic predisposition to develop autoimmune disease, as well as in healthy animals, results in SS-like disease. The NZB/WF1 strain is an sSS model that develops spontaneous lupus and SS [37]. Studies in the SMG of these animals revealed that TLR3 is expressed in the salivary epithelium, both in acinar tissue, ducts, and in the granular convoluted tubules (the segment of the duct system situated between the striated and intercalated ducts in rodents) [38, 39]. When NZB/WF1 mice were given a synthetic TLR3 agonist (polyinosinic:polycytidylic acid (poly(I:C)), they developed SS-like disease at an accelerated rate [39, 40]. Furthermore, TLR3 agonism upregulated type I interferon (*IFNβ*) and IFN-responsive genes and inflammatory mediators in SMG tissue, including *IL-6*, *IL-1β*, and *CCL5* [39, 40]. Interestingly, saliva production was reduced significantly one week post-treatment, despite the fact that the SMG histology remained normal in appearance [39]. Further work demonstrated that IFN and IL-6 are crucial for loss of salivary flow, as *IFNar^−/−^* and *IL-6^−/−^* mice were protected from salivary hypofunction following poly(I:C) administration [41]. Thus, poly(I:C) mediates salivary disease by induction of both type I IFN and IL-6.

Significantly, TLR activation in healthy mice also leads to the development of SS. Indeed, C57BL/6 mice given the TLR agonists lipopolysaccharide (LPS) or poly(I:C) display loss of salivary and tear flow and increased expression of proinflammatory cytokines in SMG and lacrimal tissue [41–43]. Additional studies implicate TLR5 in SS pathogenesis. C57BL/6 animals treated with the flagellar filament structural protein FliC, a TLR5 agonist, develop salivary inflammation [44]. Moreover, these mice display elevated levels of inflammatory mediators in sera and heightened IgG and anti-SSA/Ro autoantibodies [44]. Thus, these studies demonstrate a role for TLR activation in SS initiation in healthy animals and also in disease exacerbation in SS-prone mice. A summary of TLR-related studies in mouse models is provided in Table 1.

## 3. TLRs Are Dysregulated in Human SS

TLR expression and function have been studied in both salivary cells and PBMCs derived from pSS patients. Studies conducted on minor salivary gland (MSG) biopsy tissue or salivary gland epithelial cells (SGECs) allow for a mechanistic understanding of salivary-specific disease events in pSS. Analysis of PBMC populations provides insight regarding systemic immune dysfunction in pSS. In the following section, we will review the literature related to TLR expression and function both in salivary tissue and in the periphery in the context of pSS.

### 3.1. Cell Surface TLRs Contribute to SS Pathogenesis

#### 3.1.1. TLR2 Mediates Inflammation in SS

TLR2 plays an important role in several different autoimmune diseases, including lupus and rheumatoid arthritis [45, 46]. TLR2 recognizes a wide range of microbial products such as peptidoglycan (PGN) from Gram-positive bacteria and bacterial lipoproteins [47]. This diverse ligand binding is due in part to the ability of TLR2 to form heterodimers with TLR1 or TLR6 in order to fine-tune its specificity [48].

TLR2 is expressed in SGECs and in MSG tissue of pSS patients [49–51], and this expression correlates with the degree of focal lymphocytic sialadenitis [49]. PGN stimulation of TLR2 results in increased expression of mediators of immune activation (ICAM-1, CD40, and MHC-1) in SGECs derived from pSS patients and controls [51]. In a corroborative study using SGECs from pSS patients, TLR2 ligation resulted in NF-*κ*B-dependent secretion of IL-15 [52]. IL-15 mediates proliferation of activated B and T cells and is pivotal in the generation and maintenance of natural killer (NK) cells [53]. Further work using SGECs derived from pSS and control MSG biopsies found that IL-15 was upregulated in these cells [54]. These data indicate that TLR2 agonism could promote both the survival and proliferation of both innate and adaptive immune cells in salivary tissue in disease.

In addition, *TLR2* levels are elevated in PBMCs from pSS patients [49]. Interestingly, TLR2 stimulation results in upregulation of *IL-17* and *IL-23* mRNA transcripts, as well as heightened secretion of IL-17 and IL-23 in PBMCs derived from pSS patients as compared to controls [49]. Secretion of both cytokines is enhanced when cells are treated concomitantly with PGN and anti-CD3 [49]. These data suggest that TLR2 signaling may be an important mechanism leading to enhanced T cell-derived IL-17 production in pSS [55].

Although the ligands that activate TLR2 in the context of SS are unknown, it is interesting to note that both TLR1 and TLR6 are expressed at high levels in the SGECs of pSS patients [49]. Since TLR2 forms heterodimers with both TLR1 and TLR6, this suggests a diverse group of ligands may activate TLR2-dependent signals in disease. Significantly, activation of pSS PBMCs with TLR2 and TLR6 ligands (PGN and zymosan, respectively) results in additive secretion of IL-23 and IL-17 [49]. Altogether, these data indicate that TLR2-dependent pathways may lead to inflammation both in exocrine tissue and in the periphery. Therefore, therapies that target TLR2 may ameliorate both local and systemic pSS disease manifestations.

#### 3.1.2. Emerging Data Suggest an Important Role for TLR4 in Human SS

TLR4 recognizes both pathogen-derived and endogenous molecules and plays an important role in the pathogenesis of several autoimmune diseases [45, 47, 56–58]. TLR4 is expressed in salivary glands, specifically in infiltrating mononuclear cells and in ductal and acinar cells [49, 59]. TLR4 expression is increased in the MSG of pSS patients, and receptor levels correlate with the degree of glandular inflammation [49, 60]. Importantly, TLR4 expressed by SGECs is functional, as stimulation with LPS results in increased expression of the immunoregulatory molecules ICAM-1, CD40, and MHC-1 [51]. Although additional work reported TLR4-mediated IL-6 production in human salivary gland (HSG) cells, this cell line is now recognized to be a HeLa derivative [59, 61].

However, recent work using the A253 salivary gland cell line established that TLR4 is upregulated in response to LPS stimulation in salivary cells [62]. Moreover, LPS stimulation results in secretion of numerous inflammatory mediators, including IL-6, IL-12, CCL5, and monocyte chemotactic protein-1 (MCP-1) [62]. Thus, current evidence suggests an important role for TLR4 in salivary inflammation in SS, and future work to understand the regulation and activation of TLR4 in the context of this disease may lead to novel ways to mitigate chronic inflammation in autoimmunity.

#### 3.1.3. Hematopoietic-Derived TLR5 May Contribute to Disease

While there is a paucity of data regarding the role of TLR5 in SS (and in autoimmunity in general), TLR5-dependent signaling pathways may contribute to disease. TLR5 recognizes flagellin, a highly conserved protein found in bacteria. TLR5 expression is decreased in PBMCs from pSS patients as compared to healthy controls [63], although the significance of this finding remains to be determined. It is interesting to speculate that TLR5 activation may promote sialadenitis and autoantibody production in pSS patients, as is suggested by studies in mice (*vide supra*), although this has not been examined in humans to date [44, 64]. Further studies of TLR5 in peripheral blood samples and salivary tissue of pSS patients may reveal an important role for this poorly understood receptor in autoimmunity.

### 3.2. Endosomal TLRs Mediate Disease in SS Patients

#### 3.2.1. TLR3 Mediates Cell Death and Inflammatory Cytokine Production in Salivary Gland Cells in SS

TLR3 binds dsRNA that is primarily produced by viruses, although this receptor also recognizes endogenous RNA released from necrotic cells in autoimmune disease [47, 48, 65]. TLR3 is expressed in SGECs, and treatment of these cells with poly(I:C) results in increased of expression of ICAM-1, CD40, and MHC-I [50, 51, 66]. In addition, stimulation of SGECs with poly(I:C) or reovirus-1 (a dsRNA virus) results in heightened BAFF secretion and pre-treatment of SGECs with chloroquine diminished this effect [50]. Of note, several studies in mice and humans demonstrate that BAFF plays a key role in SS pathogenesis, primarily by inducing B cell hyperactivity [67–72]. These studies suggest that TLR3 ligation of salivary cells activates both adaptive and innate immunity.

TLR3 agonism also contributes to SGEC apoptosis in SS. Accordingly, SGECs from pSS patients stimulated with poly(I:C) undergo anoikis, a process of programmed cell death in which death is triggered by loss of normal cell attachment to the extracellular matrix (ECM) [73]. Significantly, SGECs derived from a healthy individual are protected from anoikis following TLR3 ligation, although the reason for this observation is poorly understood [73]. In a corroborative study, TLR3 agonism induced apoptosis in pSS SGECs through upregulation of RIPK3, p-FADD, and cleaved caspase-8 [74]. While apoptosis normally does not induce an immune response in healthy individuals, SS patients exhibit deficient clearance of apoptotic debris that could serve as a nidus of inflammation in disease [75].

In addition to apoptosis, TLR3 ligation in salivary tissue regulates the SS autoantigens SSA (*Ro52* and Ro60) and SSB (La) [76]. Specifically, treatment of SGECs from control and pSS patients with poly(I:C) causes redistribution of these proteins in the nucleus and increases expression of *Ro52* transcripts. Stimulation of SGECs with poly(I:C) also results in the dysregulation of interferon regulatory factors (IRFs) and elevated IFN*β* levels, similar to observations in NZB/WF1 mice (*vide supra*) [39]. Significantly, neutralization of IFN*β* diminished poly(I:C)-induced upregulation of *Ro52* mRNA and also inhibited autoantigen redistribution [76]. Taken together, these data suggest that TLR3 signaling cascades upregulate *Ro52* and induce apoptosis of salivary epithelium, thereby releasing autoantigens that drive immune hyperactivity in SS. Therefore, TLR3 has profound effects on salivary epithelium, as TLR3 agonists promote expression of costimulatory molecules, cytokine secretion, anoikis, and autoantigen expression within these cells.

#### 3.2.2. Activation of TLR7, TLR8, and TLR9 Contributes to Immune Dysregulation in SS

TLR7 and TLR8 are expressed in salivary tissue and in the periphery, and several studies demonstrate that both receptors are elevated in the context of SS [50, 63, 77–79]. Significantly, data suggest that TLR7 activation contributes to the etiopathogenesis of SS. TLR7-stimulated B cells from pSS patients secrete increased levels of IFN*α* as compared to healthy control B cells [80]. Moreover, stimulation of naïve B cells from pSS patients with a TLR7 agonist causes increased plasma cell differentiation and class switching [81]. Finally, studies using monocyte-derived DCs (moDCs) reveal that cells isolated from pSS patients exhibit enhanced maturation following stimulation with a TLR7/8 agonist (CL097) as compared to those from healthy controls [82]. Taken together, these data demonstrate that pSS patients display elevated TLR7 expression and hyperresponsiveness to TLR7/8 ligands and this likely plays an important role in the chronic inflammatory landscape observed in SS patients.

While the underlying reasons for this are unknown at present, it is intriguing to speculate that the heightened TLR7 activation in SS may be due to X chromosome gene dosage effects. As mentioned previously, SS occurs more commonly in women than in men [6]. In order to compensate for the presence of two X chromosomes in females, either the maternally- or paternally-derived X chromosome is randomly silenced, a process called X chromosome inactivation (XCI). However, this epigenetic change is not 100% effective, as 15% of genes expressed by both chromosomes exhibit altered expression [83]. A recent study using gene expression datasets found upregulation of 58 X chromosome genes, including 22 genes previously shown to escape XCI, in SS patient salivary glands [84]. Of note, TLR7 is found on the X chromosome and escapes XCI in immune cells [85]. Thus, there is a potential for females to have an increased TLR7 copy number, leading to subsequent TLR7 hypersensitivity. Indeed, pSS patients exhibit increased TLR7 expression and responsiveness [77, 78, 80, 84] and this is likely mediated, at least in part, by improper gene silencing [84].

Finally, several studies have focused on TLR9 in SS, as TLR9 levels are elevated in both MSG and parotid tissues in disease [78, 79]. While one study reported increased *TLR9* expression in PBMCs [78], others report that expression is decreased in both PBMCs and monocytes from pSS patients [63, 77]. Differences are also observed in TLR9 responsiveness, as secretion of proinflammatory mediators, plasma cell differentiation, and class switch recombination were increased following TLR9 ligation in B cells derived from pSS patients as compared to those from controls [80, 81]. Of note, expression of CD80 and CD25 was diminished as compared to healthy controls [80]. As mentioned above, TLR9 plays a protective role in lupus [31] and emerging work suggests that TLR9 activation also ameliorates disease in SS [30]. Although there are few studies regarding the role of TLR9 in SS, it is possible that TLR9 upregulation in glandular tissue may promote resolution of inflammation, while the decreased expression in the periphery may lead to immune activation. Therefore, further studies are required to understand how TLR9 is regulated in SS and how TLR9 activation modulates disease pathogenesis.

### 3.3. TLR-Dependent Signaling Molecules Are Dysregulated in SS

In addition to TLRs themselves, many TLR-dependent signaling molecules show altered expression in pSS. Accordingly, *MyD88* is upregulated in both monocytes and plasmacytoid DCs (pDCs) derived from pSS patients who demonstrate an IFN signature [77]. In addition, *PKR* (*EIF2AK2*), which is activated by TLR4 through a MyD88-independent pathway, is increased in monocytes from IFN-positive pSS patients as compared to those who are IFN-negative and healthy controls [77]. Moreover, *RSAD2*/*viperin* and *STAT1* were both increased in IFN-positive pSS patients as well [77]. STAT1 is downstream of TLR2, TLR4, TLR7, and TLR9 [77, 82, 86], while RSAD2 is induced by TLR3 and TLR7 [77, 87, 88]. Thus, evidence points to dysregulation of both TLRs and TLR-dependent signaling intermediates in SS. A summary of studies detailing TLR dysregulation in salivary tissue (Table 2) and in the hematopoietic compartment in pSS patients (Table 3) is provided.

## 4. Emerging Data Identify Novel Ligands That Likely Mediate Chronic Inflammation in SS

**Figure 1:**
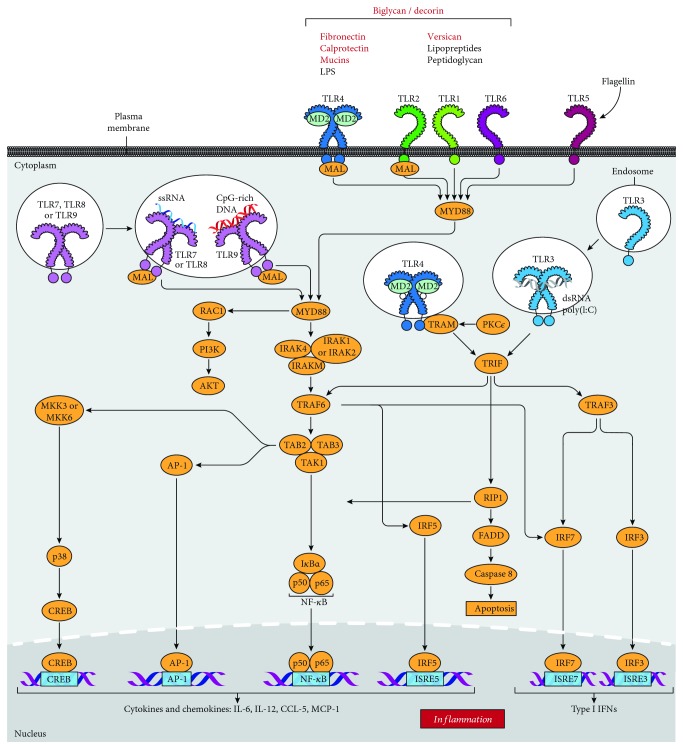
Overview of TLR signaling cascades in SS. TLRs activated in SS and downstream signaling cascades are shown. DAMPs implicated in SS are labeled in red.

In pSS, the ligands that activate TLRs remain unknown. While PAMPs may play a role in disease pathogenesis, it is possible that endogenous DAMPs may activate TLRs, as is the case for other autoimmune diseases [58, 89, 90]. DAMPs are a diverse group of stimuli that include ECM molecules, RNA and DNA, and saturated fatty acids [58, 90, 91]. DAMP-induced inflammation is “sterile,” as it is caused by host-derived molecules that normally do not elicit an immune response. However, under conditions of tissue damage, these endogenous mediators are released in soluble form, allowing for activation of host receptors [90, 92]. Many different TLRs are activated by numerous, partially overlapping DAMPs [90]. Evidence in both SS models and patients suggests that DAMPs may contribute to disease [93–95], although more work is needed to understand the role of DAMP-derived inflammation in SS. A summary of DAMPs identified in SS that are known to activate TLRs is provided in Table 4, and the signaling cascades activated by DAMPs are shown in Figure 1.

## 5. Inhibition of TLR-Dependent Signaling Mitigates Human Autoimmunity

Therapeutics that target TLR signaling pathways are currently being tested for the treatment of several different autoimmune diseases [62, 89, 90, 96–100]. Small molecule inhibitors of interleukin-1 receptor-associated kinase 4 (IRAK4) were recently developed that block collagen-induced arthritis in mice [101]. In addition, a high-throughput small-molecule screening approach identified an inhibitor that blocks the binding of TRAF6 to the 2-conjugating enzyme ubiquitin-conjugating enzyme E2N, thereby preventing ubiquitination that is required for transduction of inflammatory signaling [102]. This molecule (termed C25-140) attenuated disease severity in mouse models of imiquimod-induced psoriasis and collagen-induced arthritis [102]. Moreover, a monoclonal antibody directed against TLR4 recently completed a phase 2 clinical trial for the treatment of rheumatoid arthritis [103]. Finally, IMO-8400, an inhibitor of endosomal TLRs (TLR7, TLR8, and TLR9), diminished clinical activity in a phase 2a, randomized, placebo-controlled trial in patients with plaque psoriasis [104].

Given the success of these targeted therapeutics in other autoimmune diseases, modulation of TLR signaling cascades may constitute a successful strategy to reduce local and systemic inflammation in SS. Drugs that inhibit DAMP-mediated TLR activation hold therapeutic promise, particularly if co-receptors or accessory molecules necessary for ECM binding are targeted [105, 106]. These therapies may be designed in such a way as to preserve the ability of the host to respond to pathogens while preventing pathogenic TLR activation by endogenous sources [90, 107]. Therapeutics that target TLR-dependent immune activation will likely result in improved management of SS.

## 6. Conclusion

In summary, the etiopathogenesis of SS remains poorly understood. While substantial evidence suggests that TLR activation is an integral part of the disease, further studies are required to elucidate the mechanisms that govern TLR regulation and activation both in exocrine tissue and in the periphery. These studies will likely lead to identification of novel mediators of inflammation and targeted therapeutic strategies to reduce the innate and adaptive immune activation that is characteristic of this debilitating disease.

## Figures and Tables

**Table 1:** Evidence for TLR activation in murine studies of SS.

Strain	Results	Refs
NOD/Lt	(i) Increased *TLR1*, *TLR2*, *TLR4*, *TLR9*, and *MyD88* expression at pre-clinical and clinical disease stages (ii) Chloroquine reduced sialadenitis and *TLR1*, *TLR2*, *TLR3*, *TLR4*, and *TLR9* expression in SMG tissue	[24]
NOD/Lt	(i) Lymphocytes expressing TLR9 are present in SMG tissue (ii) PBMCs coexpressing TLR9 and p-p38 MAPK are elevated in NOD/Lt animals at 5, 8, and 10 weeks of age compared to BALB/c controls	[29]
NOD	(i) TLR9 ligation increased salivation	[30]
C57BL/6.NOD-*Aec1Aec2*	(i) *TLR3*, *TLR7*, and downstream signaling intermediates are elevated at pre-clinical disease time point in SMG tissue	[33]
NOD.B10	(i) *MyD88*-deficient females are protected against local and systemic pSS manifestations	[36]
NZB/WF1	(i) TLR3 agonism with poly(I:C) increased *IFNβ*, *Mx-1*, *PRKR*, *IF144*, *IL-6*, *TNFα*, and *CCL5* in salivary tissue and resulted in loss of salivation	[39]
NZB/WF1	(i) Poly(I:C) upregulated *CCL2*, *CCL3*, *CCL4*, *CCL7*, *CCL11*, *CCL12*, *CXCL10*, and *Cxcl13* in SMG tissue (ii) Poly(I:C) induced robust and accelerated salivary inflammation and diminished saliva production	[40]
C57BL/6	(i) Poly(I:C) treatment caused reduced salivation and increased *IL-6*, *IL-10*, and *IL-*27p28 in SMG tissue	[41]
C57BL/6	(i) Poly(I:C) induced upregulation of chemokines in lacrimal tissue, dacryoadenitis, and reduced tear production	[43]
C57BL/6	(i) LPS treatment induced sialadenitis, increased *TNFα*, *IFNβ*, IFN*γ*, and *IL-6* in SMG tissue, and caused hyposalivation	[42]
C57BL/6	(i) Flagellin caused salivary inflammation, increased inflammatory cytokines and chemokines in sera, and autoantibodies	[44]

**Table 2:** Aberrant TLR expression and activation in salivary tissue in SS.

Tissue/cell type	TLR	Results	Refs
pSS SGECs	1	(i) Increased gene expression compared to controls	[51]
Healthy and pSS SGECs	2	(i) TLR2 is expressed in SGECs (ii) Increased protein expression following treatment with PGN (iii) Upregulation of immune-activating molecules following TLR2 ligation (iv) TLR2 activation induced IL-15 secretion	[50] [51] [66] [52]
pSS MSG biopsies	2	(i) Increased protein expression compared to controls that correlates with the degree of salivary inflammation	[49]
Healthy and pSS SGECs	3	(i) TLR3 is expressed (ii) Elevated gene expression compared to controls (iii) Increased protein expression following treatment with poly(I:C) (iv) Upregulation of immune-activating molecules following TLR3 ligation	[50] [51] [66] [74]
Healthy SGECs	3	(i) Induction of BAFF mRNA and protein secretion by the TLR3 agonists poly(I:C) and a dsRNA virus	[50]
Healthy and pSS SGECs	3	(i) Poly(I:C) treatment induced expression and activation of apoptosis-related signaling intermediates and anoikis	[74] [66] [73]
Healthy and pSS MSGs	3	(i) Increased protein expression of TLR3 signaling intermediate RIPK3 kinase in pSS tissue	[74]
SGECs	3	(i) Poly(I:C) treatment upregulated *Ro52*/TRIM21 mRNA, resulting in redistribution in nucleus	[76]
pSS MSG biopsies	4	(i) Increased protein expression compared to controls that correlates with the degree of salivary inflammation	[49]
Healthy and pSS SGECs	4	(i) Elevated gene expression compared to controls (ii) Increased protein expression following LPS stimulation (iii) Upregulation of immune-activating molecules following TLR4 ligation	[51]
A253 cells	4	(i) Increased protein expression following LPS stimulation (ii) LPS-induced secretion of proinflammatory mediators	[62]
pSS MSG biopsies	6	(i) Increased protein expression compared to controls that correlates with the degree of salivary inflammation	[49]
Healthy SGECs	7	(i) TLR7 is expressed	[50]
pSS MSG biopsies	7	(i) TLR7 is expressed	[77]
Control and pSS parotid biopsies	7, 9	(i) Elevated protein expression	[78]
Healthy and pSS MSG biopsies	8, 9	(i) Elevated gene expression	[79]

**Table 3:** Systemic TLR dysregulation in human SS.

Cell type	TLR	Results	Refs
PBMCs	2	(i) PBMCs from pSS patients are more responsive to TLR2, TLR4, and TLR6 agonists than those from healthy controls (ii) TLR2, TLR4, and TLR6 agonists show additive effect in induction of IL-23 and IL-17 secretion in cultured PBMCs from pSS patients	[49]
PBMCs	5	(i) Reduced protein expression	[63]
PBMCs	7	(i) Increased gene expression	[78]
PBMCs	7	(i) Increased protein expression	[63]
B cells	7	(i) Stimulation with TLR7 agonist (CL264) causes elevated IFNα secretion	[80]
CD14+ monocytes	7	(i) Increased expression of *TLR7* in pSS patients with a positive type I IFN signature	[77]
PBMCs	8	(i) Elevated gene expression	[63]
moDCs	7/8	(i) Increased maturation in moDCs derived from pSS patients following stimulation with TLR7/8 agonist (CL097)	[82]
B cells	7, 9	(i) Stimulation of naïve B cells from pSS patients with TLR7 (imiquimod) or TLR9 (CpG) agonists causes increased plasma cell differentiation and class switching compared to controls	[81]
CD14+ monocytes	9	(i) Decreased *TLR9* expression in monocytes from both type I IFN-positive and IFN-negative pSS patients	[77]
PBMCs	9	(i) Decreased gene expression	[63]
PBMCs	9	(i) Increased gene expression	[78]
PBMCs	9	(i) Enhanced secretion of IL-8, IL-15, IL-1RA, MCP-1, and IL-2R upon stimulation with TLR9 agonist CpG (ii) TLR9 agonist (CpG) decreased CD80 and CD25 expression	[80]

**Table 4:** Summary of putative DAMP/TLR interactions in SS.

DAMP	TLR	Refs
(i) Biglycan and decorin are degraded by saliva from NOD.B10 mice (ii) Versican is increased in SGEC lines from SS patients	TLR2 and TLR4 TLR2/6 heterodimer	[95] [108]
(i) Fibronectin is dysregulated in salivary tissue from SS mice and upregulated in saliva from SS patients (ii) Calprotectin (S100A8/A9) levels are elevated in sera, salivary tissue, and saliva of pSS patients (iii) MUC5B and MUC7 are mislocalized in the ECM of pSS patients	TLR4	[109] [110] [95] [111] [112] [113] [114] [115]
(i) Long interspersed nuclear element 1 (LINE-1) is increased in MSG tissue from SS patients	TLR 7/8	[116] [117]
